# Modeling the Dependency Structure of Integrated Intensity Processes

**DOI:** 10.1371/journal.pone.0134992

**Published:** 2015-08-13

**Authors:** Yong-Ki Ma

**Affiliations:** Department of Applied Mathematics, Kongju National University, Gongju-si, Chungcheongnam-do, Republic of Korea; Southwest University, CHINA

## Abstract

This paper studies an important issue of dependence structure. To model this structure, the intensities within the Cox processes are driven by dependent shot noise processes, where jumps occur simultaneously and their sizes are correlated. The joint survival probability of the integrated intensities is explicitly obtained from the copula with exponential marginal distributions. Subsequently, this result can provide a very useful guide for credit risk management.

## Introduction

Modeling and evaluation of credit risk have been popular subjects for the last decade. They have become crucial but complicated mathematical issues. Such problems are often subtle because of the complex structures of credit risk and the correlation structures of the overall economy. It is well known that there are two types of mathematical models of credit risk: the structural approach and the intensity-based approach (see Ma and Kim [1] for a general reference). Our current paper concerns the latter.

Since Jarrow and Turnbull [2], one credit risk modeling development has been based on the default intensity of a Poisson process. The Poisson process counts the number of events and the time that these events occur in a given time interval. Compared to the Poisson process, a key feature of a Cox process (see Cox [3] and Lando [4]), also known as a doubly stochastic Poisson process, is that its arrival rate is also stochastic. It is well known that the Cox process can be applied in diverse areas such as insurance, finance, neuroscience, astrophysics etc. The properties of the Cox process are described in detail in Grandell [5]. One of the intensity processes within the Cox process which can be used to measure the impact of rare events is the shot noise process (refer to Klüppelberg and Mikosch [6]).

Joint events impact financial markets significantly because of a disproportionately large amount of joint defaults by different counterparties. One of the best ways to understand the dependence structure of joint defaults is by using copulas. A copula is a function that links univariate distributions on [0, 1]^*n*^ to a joint multivariate distribution on [0, 1]. The existence of such a function is guaranteed by the well-known Sklar theorem (See Nelsen [7] for more details). Choe et al. [8] firstly developed a theoretical framework addressing the joint distribution and provided a general equation for time-dependent copulas related to stochastic processes.

A copula approach has been developed to deal with default correlations. Li [9] introduced the one-factor Gaussian copula and showed how to build a multivariate distribution of survival times for the valuation of credit derivatives. To relax the assumption of the Gaussian distribution in the one-factor Gaussian copula, student t-copula [10, 11] is commonly used. Also, as shown by Ma and Kim [12], the copula approach enables modeling of default correlation under an intensity-based framework, where the default intensities are driven by dependent jumps. Consequently, given by an intensity-based credit risk model, we focus on the dependence structure of intensities via copula in this study.

In our previous study, we derived a generalized theoretical result for the joint Laplace transform under the Farlie–Gumbel–Morgenstern (FGM) copula, and then applied this to credit default swap (CDS) rates. To extend the dependency structure of the default intensities, we replace the FGM copula by the extended FGM copula (refer to [13]). Thus, we obtain explicitly the joint Laplace transform of integrated intensities, which promptly provides us with the joint survival (or default) probability, by using the copula with exponential marginal distributions. This is then applied to the pricing of CDS rates. Our numerical study shows that the CDS rate has a diverse dependency structure from a financial point of view, depending on the chosen parameters.

The remainder of this paper is structured as follows. In Section 2, we describe the shot noise intensity processes and the extended FGM copula with exponential marginal distributions, respectively. We calculate the joint survival probability of the integrated intensities in Section 3. The relevant jointly asymptotic distributions are obtained in Section 4. In Section 5, we numerically investigate the functional behavior of the CDS rates in terms of the relevant parameters. We present our concluding remarks in Section 6.

## Problem formulation

**Definition 1** Let (Ω, 𝓕, ℙ) be a probability space, filtered by a nondecreasing right-continuous family {𝓕_*t*_, *t* ∈ [0, *T*]} of sub-*σ*-fields of 𝓕. Let *N*_*t*_ be a Poisson process adapted to 𝓕. And put *λ*_*t*_ be a non-negative stochastic process independent of *N*_*t*_, adapted to 𝓕 and assumed to be right-continuous such that (i.e. $\int_{0}^{t}\lambda_{s}ds<\infty$ almost surely). If for all 0 ≤ *t*_1_ ≤ *t*_2_ and *u* ∈ ℝ

$$\begin{array}{c} E[e^{iu(N_{t_{2}}-N_{t_{1}})}|F_{t_{2}}^{\lambda }]=\exp[\left(e^{iu}-1\right)\int_{t_{1}}^{t_{2}}\lambda_{s}ds], \end{array}$$ (1)

then *N*_*t*_ is call a Cox process with intensity *λ*_*t*_, where ${𝓕}_{t}^{\lambda }=\sigma \{\lambda_{s};s\leq t\}$. Recall that Eq (1) satisfies

$$\begin{array}{c} P\left[N_{t_{2}}-N_{t_{1}}=k|\lambda_{s};t_{1}\leq s\leq t_{2}\right]=\frac{{\left(\int_{t_{1}}^{t_{2}}\lambda_{s}ds\right)}^{k}}{k!}\exp(-\int_{t_{1}}^{t_{2}}\lambda_{s}ds). \end{array}$$ (2)

Consider the process $\Lambda_{t}=\int_{0}^{t}\lambda_{s}ds$, then from Eq (2) we can solve that

$$\begin{array}{c} E[\theta^{N_{t_{2}}-N_{t_{1}}}]=E[e^{-\left(1-\theta \right)\left(\Lambda_{t_{2}}-\Lambda_{t_{1}}\right)}]. \end{array}$$ (3)

Eq (3) implies that the problem of finding the probability generating function of *N*_*t*_ is equal to the problem of finding the moment generating function of Λ_*t*_.

A CDS is a financial instrument for swapping the credit risk. The protection buyer of a CDS pays a premium for effectively insuring against a debt default. He receives a lump sum payment if the debt instrument is defaulted. The protection seller of a CDS receives regularly payments from the protection buyer. If the debt instrument defaults they have to pay the agreed amount to the protection buyer. Then, the credit risk of the reference entity is transferred from the protection buyer to the protection seller. Especially, in the worst case about the protection buyer, the reference entity and the protection seller are influenced by common external events. This paper therefore study the issue of the default correlation between protection seller and reference entity.

The quantitative modeling for credit risk is difficult because default events are rare and involve significant losses. In financial market, the default events can be affected by the external events, such as the subprime crisis of 2007 and firms’ bankruptcy. To capture the effects of external events, we consider the default intensities within the Cox processes. Intensity processes, which are affected by the external events, can give rise to default events. Generally, these external events result in a simultaneous positive jump in intensity processes. As time goes by, these intensity processes decrease to prevent default events after the arrival of an external event; the decreases continue until another external event occurs. To model this, we consider the default intensity processes as shot noise processes of the following form:

$$\begin{array}{ccc} d\lambda_{t}^{\left(s\right)}\;\; & = & \;\;-\delta^{\left(s\right)}\lambda_{t}^{\left(s\right)}dt+dC_{t}^{\left(s\right)},\;\;\;\;\;\;\;\;\;\;C_{t}^{\left(s\right)}:=\sum_{j=1}^{M_{t}}X_{j}^{\left(s\right)},\\ \;\;d\lambda_{t}^{\left(re\right)}\;\; & = & \;\;-\delta^{\left(re\right)}\lambda_{t}^{\left(re\right)}dt+dC_{t}^{\left(re\right)},\;\;\;\;\;\;C_{t}^{\left(re\right)}:=\sum_{j=1}^{M_{t}}X_{j}^{\left(re\right)},\\ d\lambda_{t}^{\left(b\right)}\;\; & = & \;\;-\delta^{\left(b\right)}\lambda_{t}^{\left(b\right)}dt+dC_{t}^{\left(b\right)},\;\;\;\;\;\;\;\;\;\;C_{t}^{\left(b\right)}:=\sum_{j=1}^{M_{t}^{{}^{\prime }}}X_{j}^{\left(b\right)}, \end{array}$$

where $\lambda_{t}^{\left(s\right)}$, $\lambda_{t}^{\left(re\right)}$, and $\lambda_{t}^{\left(b\right)}$ are the default intensity processes of the protection seller, the reference entity, and the protection seller in CDS, respectively, *δ* ^(*s*)^ ≥ 0, *δ* ^(*re*)^ ≥ 0, and *δ* ^(*b*)^ ≥ 0 are constants, and $C_{t}^{\left(s\right)}$, $C_{t}^{\left(re\right)}$, and $C_{t}^{\left(b\right)}$ are pure-jump processes. $\left\{X_{j}^{\left(s\right)},X_{j}^{\left(re\right)}\right\}$ is a sequence of dependent and identically distributed random variables with distributions *F* ^(*s*)^(*x* ^(*s*)^) and *F* ^(*re*)^(*x* ^(*re*)^) and $\left\{X_{j}^{\left(b\right)}\right\}$ is a sequence of independent and identically distributed random variables with distribution *F* ^(*b*)^(*x* ^(*b*)^). The point processes *M*_*t*_ and $M_{t}^{\prime }$ represent the total number of events up to time *t* with frequency *ρ* and *ρ*′, respectively. We assume that *M*_*t*_ and $M_{t}^{\prime }$ are independent of $\left\{X_{j}^{\left(s\right)},X_{j}^{\left(re\right)}\right\}$ and $\left\{X_{j}^{\left(b\right)}\right\}$, respectively. Note that shot noise intensity processes have the common point process *M*_*t*_ to model simultaneous positive jumps between the protection seller and reference entity.

To model the problem of the correlation between jump sizes for the protection seller and reference entity, we use the extended FGM copula, developed by Huang and Kotz [14], in the following form:

$$\begin{array}{c} C\left(u,v\right)=uv\left\{1+\theta {\left(1-u\right)}^{p}{\left(1-v\right)}^{p}\right\}, \end{array}$$

where *p* > 0, ∣*θ*∣ ≤ 1, *u* ∈ [0, 1], and *v* ∈ [0, 1]. Note that the range of *θ* contains the interval [−1, 1] for all *p*. The extended FGM copula has a wider range for *θ* than the FGM copula. To derive a closed-form expression of the joint Laplace transform, we assume that the marginal distributions have the following exponential forms:

$$\begin{array}{c} F^{\left(s\right)}\left(x^{\left(s\right)}\right)=1-e^{-\alpha x^{\left(s\right)}},\;\;\;F^{\left(re\right)}\left(x^{\left(re\right)}\right)=1-e^{-\beta x^{\left(re\right)}},\;\;\;F^{\left(b\right)}\left(x^{\left(b\right)}\right)=1-e^{-\gamma x^{\left(b\right)}}, \end{array}$$

where *α* > 0, *β* > 0, and *γ* > 0. Then, the extended FGM copula with exponential marginal distributions, used to model simultaneous positive jumps between the reference entity and the protection seller, is given by

$$\begin{array}{c} C\left(F^{\left(s\right)}\left(x^{\left(s\right)}\right),F^{\left(re\right)}\left(x^{\left(re\right)}\right)\right)=\left(1-e^{-\alpha x^{\left(s\right)}}\right)\left(1-e^{-\beta x^{\left(re\right)}}\right)\left(1+\theta e^{-p\alpha x^{\left(s\right)}}e^{-p\beta x^{\left(re\right)}}\right). \end{array}$$ (4)

## The joint survival probability

In the following, we derive the joint survival probability of the shot noise intensity processes with a extended FGM copula. The piecewise deterministic Markov process (PDMP) theory developed by Davis [15] is a powerful mathematical tool in this context. Using the theory, we derive the joint Laplace transform of the distribution of the vector $\left(\Lambda_{t}^{\left(s\right)},\Lambda_{t}^{\left(re\right)}\right)$ that provides us with the joint survival (or default) probability, where $\left(\Lambda_{t}^{\left(s\right)},\Lambda_{t}^{\left(re\right)}\right)$, where

$$\begin{array}{c} \Lambda_{t}^{\left(s\right)}=\int_{0}^{t}\lambda_{s}^{\left(s\right)}ds\;\;\;\;\;\text{and}\;\;\;\;\;\Lambda_{t}^{\left(re\right)}=\int_{0}^{t}\lambda_{s}^{\left(re\right)}ds. \end{array}$$

By means of the PDMP theory and the definition of the shot noise process, the joint process $\left(\Lambda_{t}^{\left(s\right)},\Lambda_{t}^{\left(re\right)},\lambda_{t}^{\left(s\right)},\lambda_{t}^{\left(re\right)},t\right)$, using the copula *C*, is known to have the infinitesimal generator 𝓐 acting on a function *f*. This infinitesimal generator is given by

$$\begin{array}{ccc} & & Af(\Lambda^{\left(s\right)},\Lambda^{\left(re\right)},\lambda^{\left(s\right)},\lambda^{\left(re\right)},t)\\ & & =\frac{\partial f}{\partial t}+\lambda^{\left(s\right)}\frac{\partial f}{\partial \Lambda^{\left(s\right)}}+\lambda^{\left(re\right)}\frac{\partial f}{\partial \Lambda^{\left(re\right)}}-\delta^{\left(s\right)}\lambda^{\left(s\right)}\frac{\partial f}{\partial \lambda^{\left(s\right)}}-\delta^{\left(re\right)}\lambda^{\left(re\right)}\frac{\partial f}{\partial \lambda^{\left(re\right)}}\\ & & \;+\rho [\int_{0}^{t}f\left(\Lambda^{\left(s\right)},\Lambda^{\left(re\right)},\lambda^{\left(s\right)}+x^{\left(s\right)},\lambda^{\left(re\right)}+x^{\left(re\right)},t\right)dC\left(F\left(x^{\left(s\right)}\right),F\left(x^{\left(re\right)}\right)\right)\\ & & \;\;\;\;\;\;\;\;\;\;\;-f(\Lambda^{\left(s\right)},\Lambda^{\left(re\right)},\lambda^{\left(s\right)},\lambda^{\left(re\right)},t)], \end{array}$$ (5)

where *F*(*x* ^(*s*)^) and *F*(*x* ^(*re*)^) denote the distributions of the jump sizes, respectively, *d*𝓒(*F*(*x* ^(*s*)^), *F*(*x* ^(*re*)^)) implies the joint probability density function of jump sizes using extended FGM copula. This is well-defined process if *f* is differentiable with respect to each independent variable and the following holds:

$$\begin{array}{ccc} & & |\int_{0}^{t}f\left(\Lambda^{\left(s\right)},\Lambda^{\left(re\right)},\lambda^{\left(s\right)}+x^{\left(s\right)},\lambda^{\left(re\right)}+x^{\left(re\right)},t\right)\;dC\left(F\left(x^{\left(s\right)}\right),F\left(x^{\left(re\right)}\right)\right)\\ & & \;\;\;\;\;-f(\Lambda^{\left(s\right)},\Lambda^{\left(re\right)},\lambda^{\left(s\right)},\lambda^{\left(re\right)},t)|<\infty . \end{array}$$

We consider shot noise intensity processes where jumps occur simultaneously and their sizes are correlated.

**Lemma 1** For arbitrary constants *γ* ^(*s*)^, *γ* ^(*re*)^, *κ* ^(*s*)^ and *κ* ^(*re*)^ such that *γ* ^(*s*)^ ≥ 0, *γ* ^(*re*)^ ≥ 0, *κ* ^(*s*)^ ≥ 0 and *κ* ^(*re*)^ ≥ 0, the process

$$\begin{array}{ccc} & & \exp\left(-\gamma^{\left(s\right)}\Lambda_{t}^{\left(s\right)}\right)\times \exp[-\{Z^{\left(s\right)}\left(\kappa^{\left(s\right)},\gamma^{\left(s\right)},t\right)\}\lambda_{t}^{\left(s\right)}]\\ & & \times \exp\left(-\gamma^{\left(re\right)}\Lambda_{t}^{\left(re\right)}\right)\times \exp[-\{Z^{\left(re\right)}\left(\kappa^{\left(re\right)},\gamma^{\left(re\right)},t\right)\}\lambda_{t}^{\left(re\right)}]\\ & & \times \exp[-\rho \int_{0}^{t}\{1-M(Z^{\left(s\right)}\left(\kappa^{\left(s\right)},\gamma^{\left(s\right)},s\right),Z^{\left(re\right)}\left(\kappa^{\left(re\right)},\gamma^{\left(re\right)},s\right)\}ds], \end{array}$$ (6)

is a martingale, where

$$\begin{array}{ccc} & & Z^{\left(s\right)}\left(\kappa^{\left(s\right)},\gamma^{\left(s\right)},s\right)=\frac{\gamma^{\left(s\right)}}{\delta^{\left(s\right)}}+(\kappa^{\left(s\right)}-\frac{\gamma^{\left(s\right)}}{\delta^{\left(s\right)}})e^{-\delta^{\left(s\right)}s},\\ & & Z^{\left(re\right)}\left(\kappa^{\left(re\right)},\gamma^{\left(re\right)},s\right)=\frac{\gamma^{\left(re\right)}}{\delta^{\left(re\right)}}+(\kappa^{\left(re\right)}-\frac{\gamma^{\left(re\right)}}{\delta^{\left(re\right)}})e^{-\delta^{\left(re\right)}s},\\ & & M\left(\varsigma ,\varphi ;s\right)=\int_{0}^{\infty }\int_{0}^{\infty }e^{-\varsigma x^{\left(s\right)}}e^{-\varphi x^{\left(re\right)}}dC\left(F\left(x^{\left(s\right)}\right),F\left(x^{\left(re\right)}\right)\right), \end{array}$$ (7)

respectively.

**Proof**. Put

$$\begin{array}{c} f=e^{-\gamma^{\left(s\right)}\Lambda_{t}^{\left(s\right)}}e^{-S^{\left(s\right)}\left(t\right)\lambda_{t}^{\left(s\right)}}e^{-\gamma^{\left(re\right)}\Lambda_{t}^{\left(re\right)}}e^{-S^{\left(re\right)}\left(t\right)\lambda_{t}^{\left(re\right)}}e^{R(t)}, \end{array}$$

for some functions *S* ^(*s*)^, *S* ^(*re*)^ and *R*. By putting this into the equation 𝓐*f* = 0, we get

$$\begin{array}{ccc} & & -\lambda^{\left(s\right)}S^{{\left(s\right)}^{\prime }}\left(t\right)-\lambda^{\left(re\right)}S^{{\left(re\right)}^{\prime }}\left(t\right)+R^{\prime }\left(t\right)-\lambda^{\left(s\right)}\gamma^{\left(s\right)}-\lambda^{\left(re\right)}\gamma^{\left(re\right)}\\ & & +\delta^{\left(s\right)}\lambda^{\left(s\right)}S^{\left(s\right)}\left(t\right)+\delta^{\left(re\right)}\lambda^{\left(re\right)}S^{\left(re\right)}\left(t\right)+\rho \left\{M\left(S^{\left(s\right)}\left(s\right),S^{\left(re\right)}\left(s\right);s\right)-1\right\}=0. \end{array}$$ (8)

This leads to

$$\begin{array}{ccc} & & S^{\left(s\right)}\left(t\right)=\frac{\gamma^{\left(s\right)}}{\delta^{\left(s\right)}}+(\kappa^{\left(s\right)}-\frac{\gamma^{\left(s\right)}}{\delta^{\left(s\right)}})e^{-\delta^{\left(s\right)}t},\\ & & S^{\left(re\right)}\left(t\right)=\frac{\gamma^{\left(re\right)}}{\delta^{\left(re\right)}}+(\kappa^{\left(re\right)}-\frac{\gamma^{\left(re\right)}}{\delta^{\left(re\right)}})e^{-\delta^{\left(re\right)}t},\\ & & R\left(t\right)=\rho \int_{0}^{t}\{1-M(\frac{\gamma^{\left(s\right)}}{\delta^{\left(s\right)}}+(\kappa^{\left(s\right)}-\frac{\gamma^{\left(s\right)}}{\delta^{\left(s\right)}})e^{-\delta^{\left(s\right)}s},\\ & & \;\;\;\;\;\;\;\;\;\;\;\;\;\;\;\;\;\;\frac{\gamma^{\left(re\right)}}{\delta^{\left(re\right)}}+(\kappa^{\left(re\right)}-\frac{\gamma^{\left(re\right)}}{\delta^{\left(re\right)}})e^{-\delta^{\left(re\right)}s}\;;s)\}ds, \end{array}$$

respectively. Then, *f* becomes a martingale because of Dynkin’s formula. Therefore, Lemma 1 is proved. ◼

**Lemma 2** Let *ξ* ^(*s*)^ ≥ 0, *ξ* ^(*re*)^ ≥ 0, *ψ* ^(*s*)^ ≥ 0 and *ψ* ^(*re*)^ ≥ 0. Then

$$\begin{array}{ccc} & & E[e^{-\xi^{\left(s\right)}\left(\Lambda {}_{t_{2}}^{\left(s\right)}-\Lambda {}_{t_{1}}^{\left(s\right)}\right)}e^{-\xi^{\left(re\right)}\left(\Lambda {}_{t_{2}}^{\left(re\right)}-\Lambda {}_{t_{1}}^{\left(re\right)}\right)}e^{-\psi^{\left(s\right)}\lambda {}_{t_{2}}^{\left(s\right)}}e^{-\psi^{\left(re\right)}\lambda {}_{t_{2}}^{\left(re\right)}}|\lambda {}_{t_{1}}^{\left(s\right)},\lambda {}_{t_{1}}^{\left(re\right)}]\\ & & =\exp[-\{\frac{\xi^{\left(s\right)}}{\delta^{\left(s\right)}}+(\psi^{\left(s\right)}-\frac{\xi^{\left(s\right)}}{\delta^{\left(s\right)}})e^{-\delta^{\left(s\right)}\left(t_{2}-t_{1}\right)}\}]\lambda {}_{t_{1}}^{\left(s\right)}\\ & & \;\times \exp[-\{\frac{\xi^{\left(re\right)}}{\delta^{\left(re\right)}}+(\psi^{\left(re\right)}-\frac{\xi^{\left(re\right)}}{\delta^{\left(re\right)}})e^{-\delta^{\left(s\right)}\left(t_{2}-t_{1}\right)}\}]\lambda {}_{t_{1}}^{\left(re\right)}\\ & & \;\times \exp[-\rho \int_{t_{1}}^{t_{2}}\{1-M(\frac{\xi^{\left(s\right)}}{\delta^{\left(s\right)}}+(\psi^{\left(s\right)}-\frac{\xi^{\left(s\right)}}{\delta^{\left(s\right)}})e^{-\delta^{\left(s\right)}\left(t_{2}-s\right)},\\ & & \;\;\;\;\;\;\;\;\;\;\;\;\;\;\;\;\;\;\;\;\;\;\;\frac{\xi^{\left(re\right)}}{\delta^{\left(re\right)}}+(\psi^{\left(re\right)}-\frac{\xi^{\left(re\right)}}{\delta^{\left(re\right)}})e^{-\delta^{\left(re\right)}\left(t_{2}-s\right)}\;;s)\}ds]. \end{array}$$ (9)

**Proof**. Let us choose *γ* ^(*s*)^, *γ* ^(*re*)^, *κ* ^(*s*)^ and *κ* ^(*re*)^ in Lemma 1 as follows:

$$\begin{array}{ccc} \gamma^{\left(s\right)} & = & \xi^{\left(s\right)},\;\;\kappa^{\left(s\right)}=\frac{\xi^{\left(s\right)}}{\delta^{\left(s\right)}}+(\psi^{\left(s\right)}-\frac{\xi^{\left(s\right)}}{\delta^{\left(s\right)}})e^{\delta^{\left(s\right)}t},\\ \gamma^{\left(re\right)} & = & \xi^{\left(re\right)},\;\;\kappa^{\left(re\right)}=\frac{\xi^{\left(re\right)}}{\delta^{\left(re\right)}}+(\psi^{\left(2\right)}-\frac{\xi^{\left(re\right)}}{\delta^{\left(re\right)}})e^{\delta^{\left(re\right)}t}, \end{array}$$

respectively. Then Lemma 1, namely, the process given by (Eq 6) is a martingale that yields the equality (Eq 9). ◼

We can get the joint Laplace transforms of the distributions $\left(\Lambda_{t}^{\left(s\right)},\Lambda_{t}^{\left(re\right)}\right)$ and $\left(\lambda_{t}^{\left(s\right)},\lambda_{t}^{\left(re\right)}\right)$ at time *t*, respectively, from Lemma 2. We merely need the joint distribution $\left(\Lambda_{t}^{\left(s\right)},\Lambda_{t}^{\left(re\right)}\right)$. However, the joint distribution $\left(\lambda_{t}^{\left(s\right)},\lambda_{t}^{\left(re\right)}\right)$ at time *t* would be used when calculating the joint Laplace transform of the distribution $\left(\Lambda_{t}^{\left(s\right)},\Lambda_{t}^{\left(re\right)}\right)$. If we set *ψ* ^(*s*)^ = *ψ* ^(*re*)^ = 0 in Lemma 2, then we get promptly

$$\begin{array}{ccc} & & E[e^{-\xi^{\left(s\right)}\left(\Lambda {}_{t_{2}}^{\left(s\right)}-\Lambda {}_{t_{1}}^{\left(s\right)}\right)}e^{-\xi^{\left(re\right)}\left(\Lambda {}_{t_{2}}^{\left(re\right)}-\Lambda {}_{t_{1}}^{\left(re\right)}\right)}|\lambda {}_{t_{1}}^{\left(s\right)},\lambda {}_{t_{1}}^{\left(re\right)}]\\ & & =\exp[-\frac{\xi^{\left(s\right)}}{\delta^{\left(s\right)}}(1-e^{-\delta^{\left(s\right)}\left(t_{2}-t_{1}\right)})\lambda {}_{t_{1}}^{\left(s\right)}]\times \exp[-\frac{\xi^{\left(re\right)}}{\delta^{\left(re\right)}}(1-e^{-\delta^{\left(re\right)}\left(t_{2}-t_{1}\right)})\lambda {}_{t_{1}}^{\left(re\right)}]\\ & & \;\times \exp[-\rho \int_{t_{1}}^{t_{2}}\{1-M(\frac{\xi^{\left(s\right)}}{\delta^{\left(s\right)}}(1-e^{-\delta^{\left(s\right)}\left(t_{2}-s\right)}),\\ & & \;\;\;\;\;\;\;\;\;\;\;\;\;\;\;\;\;\;\;\;\;\;\;\frac{\xi^{\left(re\right)}}{\delta^{\left(re\right)}}(1-e^{-\delta^{\left(re\right)}\left(t_{2}-s\right)})\;;s)\}ds]. \end{array}$$ (10)

If we set *ξ* ^(*s*)^ = *ξ* ^(*re*)^ = 0 in Lemma 2, then it follows immediately that

$$\begin{array}{ccc} & & E[e^{-\psi^{\left(s\right)}\lambda_{t_{2}}^{\left(s\right)}}e^{-\psi^{\left(re\right)}\lambda_{t_{2}}^{\left(re\right)}}|\lambda_{t_{1}}^{\left(s\right)},\lambda_{t_{1}}^{\left(re\right)}]\\ & & =\exp[-\psi^{\left(s\right)}e^{-\delta^{\left(s\right)}\left(t_{2}-t_{1}\right)}\lambda_{t_{1}}^{\left(s\right)}]\times \exp[-\psi^{\left(re\right)}e^{-\delta^{\left(re\right)}\left(t_{2}-t_{1}\right)}\lambda_{t_{1}}^{\left(re\right)}]\\ & & \;\times \exp[-\rho \int_{t_{1}}^{t_{2}}\{1-M(\psi^{\left(s\right)}e^{-\delta^{\left(s\right)}\left(t_{2}-s\right)},\psi^{\left(re\right)}e^{-\delta^{\left(re\right)}\left(t_{2}-s\right)}\;;s)\}ds]. \end{array}$$ (11)

**Theorem 1** If one uses the extended FGM copula (Eq 4), then the joint Laplace transform of the distribution $\left(\Lambda_{t}^{\left(s\right)},\Lambda_{t}^{\left(re\right)}\right)$ is given by

$$\begin{array}{ccc} & & E[e^{-\xi^{\left(s\right)}\left(\Lambda {}_{t_{2}}^{\left(s\right)}-\Lambda {}_{t_{1}}^{\left(s\right)}\right)}e^{-\xi^{\left(re\right)}\left(\Lambda {}_{t_{2}}^{\left(re\right)}-\Lambda {}_{t_{1}}^{\left(re\right)}\right)}|\lambda {}_{t_{1}}^{\left(s\right)},\lambda {}_{t_{1}}^{\left(re\right)}]\\ & & =\exp[-\frac{\xi^{\left(s\right)}}{\delta^{\left(s\right)}}(1-e^{-\delta^{\left(s\right)}\left(t_{2}-t_{1}\right)})\lambda {}_{t_{1}}^{\left(s\right)}]\times \exp[-\frac{\xi^{\left(re\right)}}{\delta^{\left(re\right)}}(1-e^{-\delta^{\left(re\right)}\left(t_{2}-t_{1}\right)})\lambda {}_{t_{1}}^{\left(re\right)}]\\ & & \;\times \exp[-\rho \int_{t_{1}}^{t_{2}}\{1-(\frac{\alpha \beta }{A^{\left(s\right)}\left(\alpha ,s\right)A^{\left(re\right)}\left(\beta ,s\right)}\\ & & \;\;\;\;\;\;\;\;\;\;\;+\frac{\theta \alpha \beta A^{\left(s\right)}\left(0,s\right)A^{\left(re\right)}\left(0,s\right)}{A^{\left(s\right)}\left(p\alpha ,s\right)A^{\left(re\right)}\left(p\beta ,s\right)A^{\left(s\right)}\left(\left(1+p\right)\alpha ,s\right)A^{\left(re\right)}\left(\left(1+p\right)\beta ,s\right)})\}ds], \end{array}$$ (12)

where

$$\begin{array}{c} A^{\left(s\right)}\left(\alpha ,s\right)=\alpha +\frac{\xi^{\left(s\right)}\left(1-e^{-\delta^{(s)s}}\right)}{\delta^{\left(s\right)}}\;\;\;\text{and}\;\;\;A^{\left(re\right)}\left(\beta ,s\right)=\beta +\frac{\xi^{\left(re\right)}\left(1-e^{-\delta^{(re)s}}\right)}{\delta^{\left(re\right)}}. \end{array}$$

**Proof**. It we apply (Eq 4) to (Eq 7), then (Eq 10) leads to (Eq 12) by a direct computation.

## The jointly asymptotic distribution

Since the infinitesimal generator (Eq 5) is time-homogeneous, we are required to find initial values. Noting that the conditional expectation in Theorem 1 is not independent of *t*_1_, let us obtain the jointly asymptotic distribution of the vector $\left(\lambda_{t}^{\left(s\right)},\lambda_{t}^{\left(re\right)}\right)$ at time *t* from (Eq 11), provided that the process started sufficiently far in the past. In this context, we interpret it as the limit when *t* → −∞ and it is called a −∞ jointly asymptotic distribution. So, if we know the distribution $\left(\lambda_{t}^{\left(s\right)},\lambda_{t}^{\left(re\right)}\right)$ at time −∞ and no information between −∞ to present time *t*, then the −∞ jointly asymptotic distribution $\left(\lambda_{t}^{\left(s\right)},\lambda_{t}^{\left(re\right)}\right)$ can be used as the distribution $\left(\lambda_{t}^{\left(s\right)},\lambda_{t}^{\left(re\right)}\right)$.

**Lemma 3** If the −∞ jointly asymptotic distribution $\left(\lambda_{t}^{\left(s\right)},\lambda_{t}^{\left(re\right)}\right)$ exists, then it is given by

$$\begin{array}{ccc} & & E[e^{-\psi^{\left(s\right)}\lambda_{t}^{\left(s\right)}}e^{-\psi^{\left(re\right)}\lambda_{t}^{\left(re\right)}}]\\ & & =\exp[-\rho \int_{-\infty }^{t}\{1-M(\psi^{\left(s\right)}e^{-\delta^{\left(s\right)}\left(t-s\right)},\psi^{\left(re\right)}e^{-\delta^{\left(re\right)}\left(t-s\right)}\;;s)\}ds]. \end{array}$$

**Proof**. As *t*_1_ → −∞, (Eq 11) implies

$$\begin{array}{ccc} & & E[e^{-\psi^{\left(s\right)}\lambda_{t_{2}}^{\left(s\right)}}e^{-\psi^{\left(re\right)}\lambda_{t_{2}}^{\left(re\right)}}]\\ & & =\exp[-\rho \int_{-\infty }^{t_{2}}\{1-M(\psi^{\left(s\right)}e^{-\delta^{\left(s\right)}\left(t_{2}-s\right)},\psi^{\left(re\right)}e^{-\delta^{\left(re\right)}\left(t_{2}-s\right)}\;;s)\}ds]. \end{array}$$

Then, the lemma follows immediately by letting *t*_2_ = *t*.

Once we have Lemma 3, we can obtain the following result for the joint Laplace transform. This is the main result.

**Theorem 2** If the vector $\left(\lambda_{t}^{\left(s\right)},\lambda_{t}^{\left(re\right)}\right)$ is ‘−∞ jointly asymptotic, then

$$\begin{array}{ccc} & & E[e^{-\xi^{\left(s\right)}\left(\Lambda {}_{t_{2}}^{\left(s\right)}-\Lambda {}_{t_{1}}^{\left(s\right)}\right)}e^{-\xi^{\left(re\right)}\left(\Lambda {}_{t_{2}}^{\left(re\right)}-\Lambda {}_{t_{1}}^{\left(re\right)}\right)}]\\ & & ={\left[\frac{\alpha \delta^{\left(s\right)}}{\alpha \delta^{\left(s\right)}+\xi^{\left(s\right)}\left\{1-e^{-\delta^{\left(s\right)}\left(t_{2}-t_{1}\right)}\right\}}\right]}^{\frac{\rho }{\delta^{\left(s\right)}}}\times {\left[\frac{\beta \delta^{\left(re\right)}}{\beta \delta^{\left(re\right)}+\xi^{\left(re\right)}\left\{1-e^{-\delta^{\left(re\right)}\left(t_{2}-t_{1}\right)}\right\}}\right]}^{\frac{\rho }{\delta^{\left(re\right)}}}\\ & & \;\times \exp[-\rho \int_{t_{1}}^{t_{2}}\{1-(\frac{\alpha \beta }{A^{\left(s\right)}\left(\alpha ,s\right)A^{\left(re\right)}\left(\beta ,s\right)}\\ & & \;\;\;\;\;\;\;\;\;\;\;+\frac{\theta \alpha \beta A^{\left(s\right)}\left(0,s\right)A^{\left(re\right)}\left(0,s\right)}{A^{\left(s\right)}\left(p\alpha ,s\right)A^{\left(re\right)}\left(p\beta ,s\right)A^{\left(s\right)}\left(\left(1+p\right)\alpha ,s\right)A^{\left(re\right)}\left(\left(1+p\right)\beta ,s\right)})\}ds]. \end{array}$$ (13)

**Proof**. Let *t*_2_ = *t*_1_ and *t*_1_ = *t*_0_ in (Eq 11), and then put *t*_0_ → −∞. If we apply (Eq 4) and (Eq 7) to (Eq 11), then we have

$$\begin{array}{c} E[e^{-\psi^{\left(s\right)}\lambda_{t_{1}}^{\left(s\right)}}e^{-\psi^{\left(re\right)}\lambda_{t_{1}}^{\left(re\right)}}]={\left(\frac{\alpha }{\alpha +\psi^{\left(s\right)}}\right)}^{\frac{\rho }{\delta^{\left(s\right)}}}{\left(\frac{\beta }{\beta +\psi^{\left(re\right)}}\right)}^{\frac{\rho }{\delta^{\left(re\right)}}} \end{array}$$ (14)

by direct computation. Now let us take the expectation of Eq (12). Then, the conditional expectation on the left-hand side (LHS) of the equation is changed into the expectation; this is the same as the LHS of (Eq 13). For the right-hand side (RHS), if we choose *ψ* ^(*s*)^ and *ψ* ^(*re*)^ as

$$\begin{array}{ccc} & & \psi^{\left(s\right)}=\frac{\xi^{\left(s\right)}}{\delta^{\left(s\right)}}(1-e^{-\delta^{\left(s\right)}\left(t_{2}-t_{1}\right)})\;\;\;\text{and}\;\;\;\psi^{\left(re\right)}=\frac{\xi^{\left(re\right)}}{\delta^{\left(re\right)}}(1-e^{-\delta^{\left(re\right)}\left(t_{2}-t_{1}\right)}), \end{array}$$

and substitute the resultant *ψ* ^(*s*)^ and *ψ* ^(*re*)^ into the expectation on the RHS of Eq (14), the we get the RHS of (Eq 13).

Note that (Eq 13) yields the joint survival (or default) probability by setting *ξ* ^(*s*)^ = *ξ* ^(*re*)^ = 1.

## Pricing CDS rates

To determine the CDS rates, we take a generalized Cox–Ingersoll–Ross model [16] as the interest rate process *r*_*t*_ for a zero-coupon default-free bond. We also use a theoretical result Eq (13) for the pricing of a zero-coupon defaultable bond with zero recovery and a deterministic payoff value of 1, paid at *t*_*k*_ when a reference entity defaults in (*t*_*k*−1_, *t*_*k*_] and the protection seller survives up to *t*_*k*_ for 0 ≤ *k* ≤ *K*. Then, the CDS rates, denoted by 𝓢, are given by

$$\begin{array}{c} S=\left(1-\pi \right)\frac{\sum_{k=1}^{k_{N}}E\left[e^{-\int_{0}^{t_{k}}r_{s}ds}|r_{0}\right]E\left[e^{-\Lambda_{t_{k-1}}^{\left(re\right)}-\Lambda_{t_{k-1}}^{\left(s\right)}}e^{-\Lambda_{t_{k}}^{\left(s\right)}-\Lambda_{t_{k-1}}^{\left(s\right)}}-e^{-\Lambda_{t_{k}}^{\left(re\right)}-\Lambda_{t_{k}}^{\left(s\right)}}|r_{0},\lambda_{0}^{\left(re\right)},\lambda_{0}^{\left(s\right)}\right]}{\sum_{n=1}^{N}(t{}_{k_{n}}-t_{k_{n-1}})E\left[e^{-\int_{0}^{t_{n}}r_{s}ds}|r_{0}\right]E\left[e^{-\Lambda_{t_{n}}^{\left(b\right)}}|\lambda_{0}^{\left(b\right)}\right]}, \end{array}$$

where *π*, *N*, and *k*_*n*_ denote the recovery rate, the number of payment dates for the protection buyer, and the dates of these payments (i.e., *t*_*k*_*n*__, *n* = 1, 2, ⋯, *N*), respectively. Refer to Ma and Kim [12].

We calculate the price of the CDS rates numerically and investigate the functional behavior of the CDS rates with respect to the relevant parameters. The parameter values used to calculate the CDS rates are

$$\begin{array}{ccc} & & \alpha =10,\;\beta =5,\;\gamma =15,\;\delta^{\left(s\right)}=0.5,\;\delta^{\left(re\right)}=0.3,\;\delta^{\left(b\right)}=0.2,\;\theta =1,\\ & & \rho =4,\;\rho^{\prime }=3,\;\pi =1/2,\;N=2,\;t_{k_{0}}=0,\;t_{k_{1}}=0.5,\;t_{k_{2}}=1,\\ & & r_{0}=0.05,\;a=0.05,\;b=0.025,\;c=1,\;\sigma =0.8. \end{array}$$

Table 1 shows that the CDS rate increases with the value of the copula parameter *p*. It also is approximately converging to 0.42 as the values of *p* are increased. In the financial market, stabler protection sellers must receive higher CDS rates, whereas protection buyers pay lower CDS rates with respect to a reference entity with a relatively high credit rating. The increasing behavior shown in Table 1 is due to the greater impact of the protection seller, but this is a minor issue because the CDS rate is monotonically increasing only slightly as *p* increases. The calculations of CDS rates caused by changes in the values of *δ* ^(*s*)^ and *α* for the protection seller and *δ* ^(*re*)^ and *β* for the reference entity are shown in Tables 2 and 3, respectively, for *p* = 0.1. Table 2 shows that the CDS rates increase when the values of *δ* ^(*s*)^ and *α* increase, respectively. However, Table 3 shows that the values of the CDS rate decrease when the values of *δ* ^(*re*)^ and *β* increase, respectively. The parameters *δ* ^(*s*)^ and *δ* ^(*re*)^ are the exponential decay rates (that is, how quickly the entity can prevent a default after the arrival of a primary event) for the protection seller and reference entity, respectively. In addition, the parameters *α* and *β* describe the magnitude of jump sizes for the protection seller and reference entity, respectively. The monotonic behavior with respect to the protection seller and reference entity shown in Tables 2 and 3, respectively, is an obvious effect from a financial point of view. Tables 2 and 3 confirm how fast the CDS rate varies. From the above investigation, we note that the CDS rate has a diverse dependency structure regarding the relevant parameters. This may be monotonic behavior from a financial point of view.

**Table 1:** CDS rates with respect to *p*.

*p*	CDS rate
0.1	0.3999
0.3	0.4171
0.5	0.4190
0.7	0.4194
1	0.4197

**Table 2:** CDS rates for protection seller.

**δ*^(*s*)^*	CDS rate	**α**	CDS rate
0.7	0.4985	9	0.3671
0.9	0.5640	12	0.4556
1.5	0.6724	15	0.5202
2.0	0.7197	20	0.5954

**Table 3:** CDS rates for reference entity.

**δ*^(*re*)^*	CDS rate	**β**	CDS rate
0.5	0.3022	7	0.3502
0.7	0.2226	10	0.2874
0.9	0.1634	13	0.2404
1.5	0.0608	20	0.1712

## Final remarks

Based on the shot noise intensity processes, we studied the important issue of default correlation in credit risk modeling. To model this, we used a multivariate shot noise intensity process, where jumps occur simultaneously and their sizes are correlated. The joint Laplace transforms of the integrated intensities, which promptly provides us with the joint survival (or default) probability, were obtained from the extended FGM copula with exponential marginal distributions. Also, we applied this result to CDS rates and then our numerical results suggested that CDS rates have a diverse dependency structure with respect to the relevant parameters from a financial point of view. Our result provides a guide for credit risk management. More comparative studies on Gaussian and Student-t copulas and applying our theoretical result to market data for calibration (refer to [13] and [17]) would be helpful to complete the present work. These remain topics for future research.
